# CryoVirusDB: An Annotated Dataset for AI-Based Virus Particle Identification in Cryo-EM Micrographs

**DOI:** 10.3390/v18020224

**Published:** 2026-02-11

**Authors:** Rajan Gyawali, Ashwin Dhakal, Liguo Wang, Jianlin Cheng

**Affiliations:** 1 Department of Electrical Engineering and Computer Science, University of Missouri, Columbia, MO 65211, USA; rgkg2@missouri.edu (R.G.); ad256@umsystem.edu (A.D.); 2 NextGen Precision Health, University of Missouri, Columbia, MO 65211, USA; 3 Laboratory for BioMolecular Structure (LBMS), Brookhaven National Laboratory, Upton, NY 11973, USA; lwang1@bnl.gov

**Keywords:** cryo-electron microscopy, AI-based virus particle picking, structural biology, viral structure, labeled dataset

## Abstract

With the advancements in instrumentation, image processing algorithms, and computational capabilities, single-particle cryo-electron microscopy (cryo-EM) has achieved atomic resolution in determining the 3D structures of viruses. The virus structures play a crucial role in studying their biological function and advancing the development of antiviral vaccines and treatments. Despite the effectiveness of artificial intelligence (AI) in general image processing, its development for identifying and extracting virus particles from cryo-EM micrographs has been hindered by the lack of manually labeled high-quality datasets. To fill the gap, we introduce CryoVirusDB, a labeled dataset containing the coordinates of expert-picked virus particles in cryo-EM micrographs. CryoVirusDB comprises 9941 micrographs from nine datasets representing seven distinct non-enveloped viruses exhibiting icosahedral or pseudo-icosahedral symmetry, along with coordinates of 339,398 labeled virus particles. It can be used to train and test AI and machine learning (e.g., deep learning) methods to accurately identify virus particles in cryo-EM micrographs for building atomic 3D structural models for viruses.

## 1. Introduction

**Figure 1:**
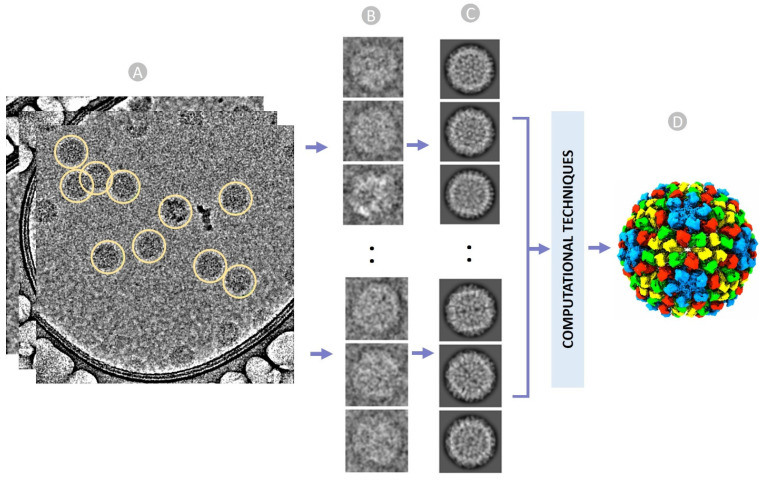
An overview of cryo-EM single particle analysis from particle selection to 3D reconstruction of virus (EMPIAR 11060). (**A**) Stack of ideal micrographs where the true virus particles are picked (encircled yellow). (**B**) Extracted virus particles from micrographs with fixed box size. (**C**) Multiple 2D classes to facilitate stack cleaning and the removal of false particles. (**D**) Reconstructed 3D structure of the virus from 2D images using a series of computational techniques. Subunits/domain regions are displayed in different colors.

Cryo-EM is a method of capturing 2D images of biological molecules and assemblies at cryogenic temperatures. Advancements in both instrumentation and computational methodologies have established cryo-EM as an essential tool for interrogating the structures and dynamics of biological macromolecular complexes including large virus particles [1,2,3,4]. Single particle experiment and data analysis in cryo-EM involve vitrifying biological specimens and collecting micrographs of individual particles in an electron microscope (Figure 1A), followed by picking and extracting particle images (Figure 1B), applying image processing for correction and alignment (Figure 1C), and performing three-dimensional (3D) reconstruction of macromolecular complexes [4,5] (Figure 1D).

In the realm of virology, cryo-EM has been instrumental in studying and determining the 3D structures and morphology of various viruses such as poliovirus, Ebola, HIV, and coronaviruses [6,7]. Particularly during the COVID-19 pandemic, cryo-EM played a pivotal role in understanding the intricate structure of the SARS-CoV-2 spike protein [8,9,10]. This knowledge has facilitated the development of highly effective vaccines. For instance, scientists have been able to design immunogens that mimic the spike protein’s shape, eliciting targeted immune responses [11,12,13]. Moreover, cryo-EM has revolutionized epitope mapping, enabling the identification of specific binding sites [14] and facilitating the exploration of antibody mutations for the rapid discovery and development of precise vaccines and antiviral treatments [15,16].

To achieve high-resolution 3D reconstructions of virus structures, the initial step of accurately recognizing and extracting virus particles from 2D image projections (micrographs) is crucial. Currently, three virus particle-picking approaches are employed: manual virus particle picking, template-based picking [17,18], and AI-based picking [19]. Manual picking is laborious and time-consuming, requiring specialized expertise for precise identification, which cannot be used by beginners. Challenges in the manual picking arise from low signal-to-noise ratios, low particle contrast, and the unpredictability of individual particle appearances due to orientation variations. Template-based virus particle picking requires users to pick some initial particles as templates for software tools to search for more particles, which suffers from the presence of ice contamination, carbon areas, and overlapping aggregated particles in micrographs. AI-based particle picking [20,21,22,23,24] has the best potential to automate the process and overcome the problems of the manual picking and template-based matching, but the development of sophisticated AI-based virus particle-picking methods is largely hindered by the lack of high-quality labeled training and test data of virus particles.

**Figure 2:**
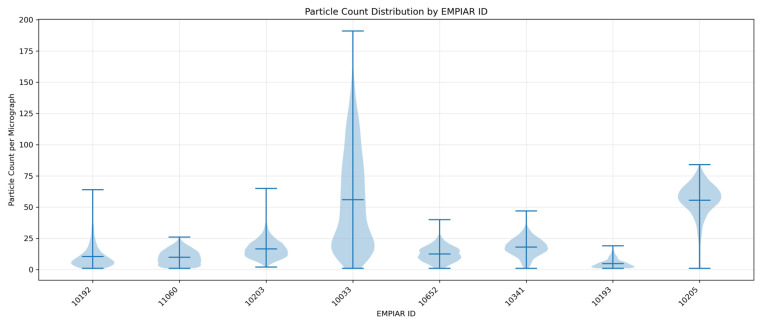
Particle count distribution across CryoVirusDB datasets. Violin plots show the distribution of particle counts per micrograph for each EMPIAR dataset. Horizontal lines indicate mean count (center), maximum count (top) and minimum count (bottom) of virus particles.

To harness the power of cutting-edge AI technologies in automatic virus particle recognition and picking, we created a comprehensive and expert-labeled dataset—CryoVirusDB [25] in this work. This open-access dataset aims to expedite the development of automated virus particle-picking workflows, and ultimately advance the viral research and the design of therapeutic interventions. CryoVirusDB includes 9941 micrographs from nine datasets representing seven distinct non-enveloped viruses with icosahedral or pseudo-icosahedral symmetry, along with coordinates of 339,398 labeled virus particles. The particle count distribution per micrograph varies considerably across datasets, as illustrated in Figure 2. The detailed statistics of CryoVirusDB are reported in Table 1 and Table 2.

## 2. Materials and Methods

### 2.1. Raw Data Acquisition and Preprocessing

The cryo-EM virus micrographs and their associated metadata were downloaded from the EMPIAR web portal [34] using Python API and FTP scripts. The comprehensive metadata include the EMPIAR ID for each cryo-EM dataset of a virus along with the corresponding identifiers such as Electron Microscopy Data Bank (EMDB) ID and Protein Data Bank (PDB) ID. Additionally, the dataset size, resolution, total number of micrographs, image specifications (size and type), pixel size, micrograph file extension, gain/motion correction file extension (if any), FTP and Globus paths for micrograph/gain files, and relevant publication information were recorded.

### 2.2. Software and Computation Tools

CryoSPARC [35] was employed for multiple stages of the particle-picking pipeline, including motion correction, patch-based contrast transfer function (CTF) estimation, interactive manual particle selection, template-based automated particle picking, two-dimensional (2D) classification, ab initio three-dimensional (3D) reconstruction, and homogeneous refinement. The software’s graphical user interface facilitated visual inspection and quality control of picked particles throughout the curation process.

For comparative validation of particle-picking quality, Topaz [22] was used as a representative artificial intelligence (AI)-based particle-picking method. Topaz was executed with default parameters to provide an unbiased comparison with our manually curated approach.

Visualization and inspection of particle stacks and 3D density maps were conducted using UCSF ChimeraX [36]. Custom Python scripts (Python version 3.8) were developed for automated data acquisition from the EMPIAR database, metadata management, and file format conversions. The Python function download_micrographs() can be used to download micrographs directly from EMPIAR website, and data format conversion functionalities can be found in convert_cs_file_to_star.py and convert_star_to_csv_file.py. Details on the usage of these files and functions can be found at https://github.com/BioinfoMachineLearning/CryoVirusDB (accessed on 1 December 2025).

## 3. Results

CryoVirusDB, a labeled dataset for AI-based training for detection of icosahedral virus particles from cryo-EM micrographs was developed. The dataset contains the cryo-EM micrographs and the associated coordinate information for training and validation of the AI models. To ensure the generalization capability of these models, we have maintained the dataset diversity by selecting 9 representative EMPIAR virus datasets with 7 unique viruses that encompass a broad range of particle sizes, shapes, density distributions, noise levels, and variations in ice thickness and carbon areas. The viruses in CryoVirusDB represent diverse taxonomic groups. Specifically, the database includes viruses from three families within the Order Picornavirales: Caliciviridae (Feline calicivirus), Picornaviridae (Human parechovirus 3, Coxsackievirus B4, and Bovine enterovirus), and Secoviridae (Cowpea mosaic virus). Additionally, it includes Nudaurelia capensis omega virus from the family Alphatetraviridae (Order Hepelivirales), and Macrobrachium rosenbergii nodavirus from the family Sarthroviridae (unassigned order) [37]. The database encompasses viruses that infect diverse hosts across multiple kingdoms, including humans (Human parechovirus 3, Coxsackievirus B4), animals (Bovine enterovirus, Feline calicivirus), arthropods (Nudaurelia capensis omega virus, Macrobrachium rosenbergii nodavirus), and plants (Cowpea mosaic virus), demonstrating the broad ecological and biological relevance of CryoVirusDB.

### 3.1. Particle-Picking Workflow

#### 3.1.1. Micrograph Import

For each EMPIAR virus dataset, cryo-EM micrographs were imported into CryoSPARC using the corresponding microscopy parameters obtained from the EMPIAR metadata. Essential acquisition parameters including pixel size (Å), accelerating voltage (kV), spherical aberration coefficient (mm), and total electron dose (e/Å^2^) were specified during the import process to ensure accurate downstream processing. The complete set of imaging parameters for all nine datasets is provided in Table 2.

#### 3.1.2. Motion Correction and Patch-Based CTF Estimation of Micrographs

In our study, we used motion-corrected micrographs in CryoSPARC [35] for patch-based Contrast Transfer Function (CTF) estimation. Since CTF functions can vary substantially among micrographs and cannot be precisely predefined, accurately identifying CTF parameters for each micrograph is crucial. This precision is necessary for proper corrections and achieving high-resolution 3D reconstructions. Two stages (estimating the CTF and correcting it) were applied to the CTF analysis.

**Figure 3:**
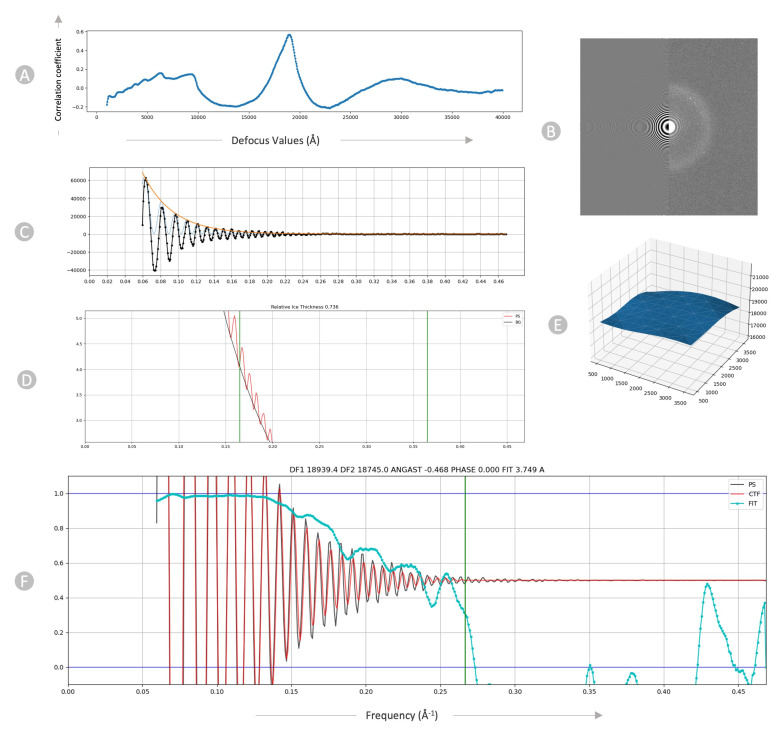
Diagnostic plots of CTF for EMPIAR 11060. (**A**) 1D search over varying defocus values. (**B**) Thon rings visible in the Fourier transform. (**C**) Fitted envelope function diagnostic plot. (**D**) Power spectrum showing the relative ice thickness. (**E**) 2D Patch result. (**F**) CTF fit plot. The frequency, measured in inverse angstroms (Å^−1^), is represented on the *X*-axis, while the correlation metric between the power spectrum (PS) and CTF value is shown on the *Y*-axis. The black line corresponds to the observed experimental power spectrum, the red line represents the calculated CTF, and the cyan line indicates the cross-correlation (FIT).

We employed the patch-based CTF tool in CryoSPARC to generate output micrographs containing information about their average defocus and the defocus landscape. Upon particle extraction, this information was automatically used to allocate a local defocus value to each particle based on its position in the landscape. The one-dimensional search across defocus values for a micrograph is shown in Figure 3A and Thon rings in the power spectra of images are shown in Figure 3B.

The plot in Figure 3C serves primarily as a verification for the successful execution of background subtraction and envelope function fitting. The *X*-axis represents frequency in inverse angstroms. The radially averaged power spectrum is depicted in black, where high values correspond to the bright portions of the Thon rings and low values to the dark regions. The orange curve represents the envelope function, aiming to model the expected falloff of Thon rings up to the Nyquist resolution, accounting for aberrations. Lastly, the fitted Contrast Transfer Function (CTF), scaled by the envelope function, is presented in blue. This oscillating plot is crucial for confirming the proper execution of background subtraction and envelope-fitting procedures. In the plot in Figure 3D, we assessed the background strength (depicted by the black line) within the area where thicker ice leads to an augmented background referred to as relative ice thickness.

The 3D surface plot (Figure 3E) shows the local defocus estimated throughout the micrograph. The surface plot in blue illustrates the defocus that has been fitted for each position along the micrograph. The x- and y-coordinates align with the micrograph’s coordinates, while the z-coordinate represents the defocus values. The X, Y, and Z axes are all expressed in Angstrom. The CTF fit plot, illustrated in Figure 3F, depicts the alignment between the simulated and observed Thon rings in the micrograph, accounting for variations in defocus and astigmatism. The cyan curve indicates the cross-correlation fit level. The CTF fit resolution (3.749 angstroms) is the resolution at which this value drops below a threshold. The vertical green line in the plot signifies the frequency at which the fit deviates from the cross-correlation threshold of 0.3, indicating a successful fit.

#### 3.1.3. Manual Particle Picking and 2D Class Formation

**Figure 4:**
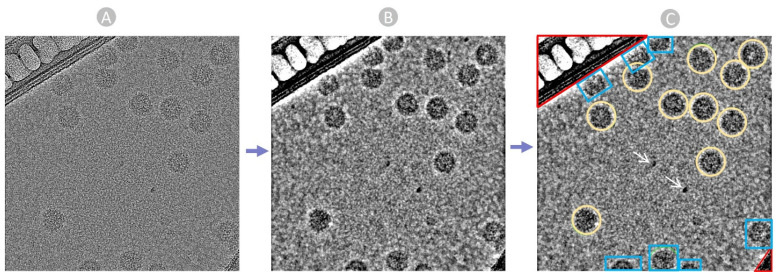
The manual picking process. (**A**) Raw micrograph obtained from EMPIAR. (**B**) Preprocessed micrograph with low-pass filter: 28 A to ease particle recognition and picking. (**C**) Manually picked true virus particle encircled in yellow, carbon region colored in red, ice patches and artifacts noted by white arrows, and cut particles in edges colored in blue.

Following the CTF estimation, we manually identified and selected true virus particles interactively from aligned and motion-corrected micrographs with the aim of generating some particle templates. We specify the particle diameter based on the virus particles’ size and shape. Picking particles directly from raw noisy micrographs is challenging (Figure 4A). So, we adjusted the ‘Contrast Intensity Override’ using a low-pass filter while inspecting micrographs to achieve the most distinct view for particle selection (Figure 4B). Figure 4C shows the picked particles for subsequent 2D alignment steps.

Manually selecting particles from raw micrographs with smaller defocus values proved to be quite challenging. To create a comprehensive set of ground-truth templates covering a broad range of defocus values, we manually picked particles from numerous micrographs exhibiting diverse defocus and CTF fit values. Given the time-intensive nature of manual picking, we chose a small subset of micrographs (around 15% of the micrographs) specifically for generating templates. The detailed information about the manually picked particles and the micrographs considered for the manual picking can be found in Supplementary Table S1.

After manually picking the virus particles, the coordinates of particle centers and the designated box size were used to extract particles from the original micrographs. During this process, the box size was defined to provide ample padding, usually ranging from 25% to 50% extra space around the particles. The manually selected particles underwent a 2D classification step, where we categorized and chose the most favorable classes. This classification step organized particles into distinct 2D classes, streamlining the cleaning of the particle stack and removal of undesirable particles. Finally, we assessed the quality of the particles and eliminated classes containing unwanted particles. The remaining particle classes were used by the template-based picking for the identification of high-quality particle classes.

#### 3.1.4. Template-Based Picking

After exporting the optimal particle classes, we employed templates created in the ‘2D Class Formation’ step in CryoSPARC. We followed an iterative approach, wherein the output from ‘template-based picking and inspection’ was once again utilized in the ‘2D Class Formation’ step to select only the high-quality 2D particles, discarding the false positives. This cycle was repeated until we obtained high-resolution particles that encompassed all possible viewing directions of the virus particle.

Using CryoSPARC’s Template Picker job, we employed the high-resolution templates to precisely pick virus particles that aligned with the geometry of the target structure. We set specific constraints, such as the particle diameter in angstrom and a minimum distance between the particles for generating templates based on the SK97 sampling algorithm [38].

#### 3.1.5. Manual Particle Inspection and Extraction

**Figure 5:**
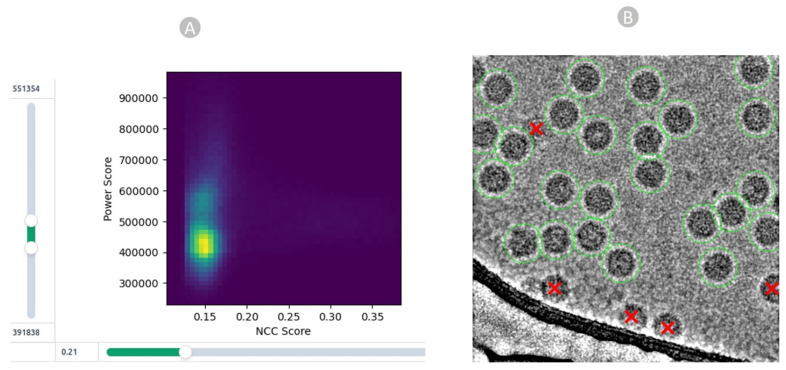
Particle quality inspection. (**A**) Particle filtration achieved by manipulating the NCC score on the *X*-axis and local power score on the *Y*-axis for EMPIAR 11060. (**B**) High-quality true virus particles (depicted by green circles) chosen through the template-based picking process and eliminated radiation-damaged, cut, and false-positive particles, represented by red cross markers.

The acquired particles above underwent manual inspection, in which we scrutinized and refined the picked particles using different thresholds. In CryoSPARC, we fine-tuned parameters such as the low-pass filter, normalized cross-correlation (NCC), and power threshold (Figure 5A) to eliminate false positives. The 2D colored histogram plots were employed to carefully analyze the median pick scores of micrographs against defocus, aiding in the extraction of coordinates for high-quality virus particles as depicted in Figure 5B.

Ultimately, we applied a 2D classification step to perform a final examination of the selected particles. The Select 2D job categorized particles into various 2D classes (usually 50 in our case), aiding in stack cleaning and the elimination of undesired particles. This process is valuable not only for assessing particle quality before entering the 3D reconstruction phase but also for qualitatively exploring the distribution of views within the dataset. Following 2D classification, certain classes are identified as “junk” classes, representing non-particle images, ice crystals, or instances of two particles being conjoined. Consequently, we filtered out the particles associated with these “junk” classes from the picked particles. More information about the overall intermediate metadata and the final set of true virus particles can be found in Supplementary Table S1.

These final true particles were exported in the form of particle stacks and star files, which include a lot of information about the particles in micrographs such as the X-coordinate, Y-coordinate, Angle-Psi, Origin X (Ang), Origin Y (Ang), Defocus U, Defocus V, Defocus Angle, Phase Shift, CTF B Factor, Optics Group, and Class Number. The star files are the widely accepted file format in cryo-EM processing software. These star files are also converted to csv files which are easy to use in python scripts and contain the necessary coordinates for generating ground truth labels while training and validating the machine learning models.

### 3.2. Data Organization of CryoVirusDB

**Figure 6:**
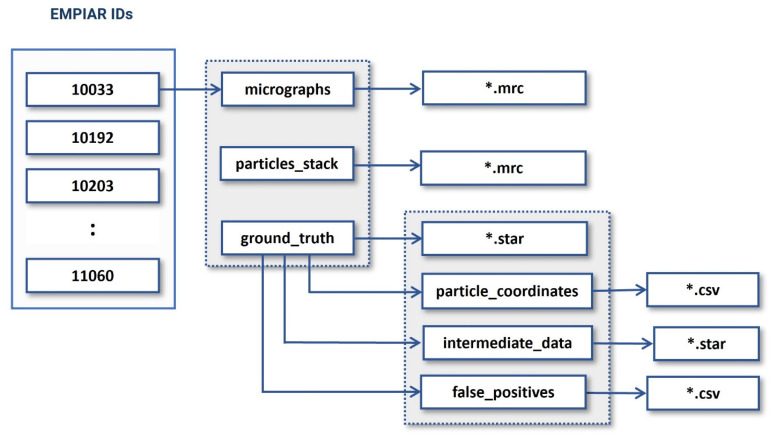
The directory structure of CryoVirusDB. The numbers in the blocks on the left side are the respective EMPIAR IDs.

CryoVirusDB includes 9 virus subsets (each including approximately 1100 cryo-EM micrographs) along with the labeled coordinates of the virus particles in the micrographs. The total size of the CryoVirusDB database is 634 GB. The organizational structure of the directories of CryoVirusDB is depicted in Figure 6.

### 3.3. Data Validation

For training machine learning models, high-quality curated data is essential. Following the data validation practices established in CryoPPP [4], we validated the quality of picked particles through both 2D classification analysis and 3D reconstruction methods. This comprehensive validation approach ensures that the particle coordinates in CryoVirusDB meet the stringent quality standards required for developing robust AI-based particle-picking algorithms.

#### 3.3.1. 2D Particle Class Validation

**Figure 7:**
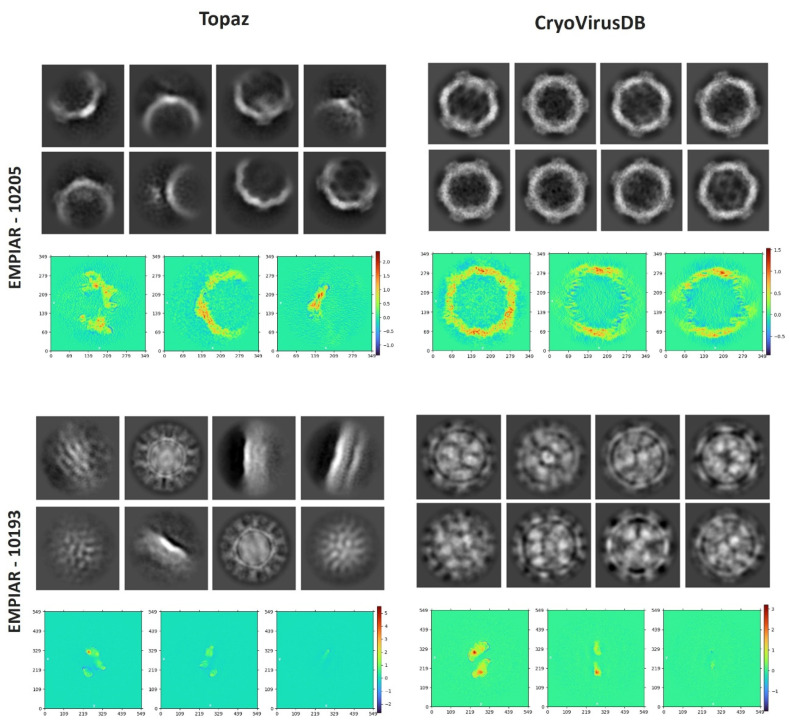
The comparison of 2D particle classification and density projections from the intermediate output of the ab initio reconstruction phase for EMPIAR 10205 and EMPIAR 10193. For each EMPIAR ID, the 2D classes are visualized at the top and the density projects are visualized at the bottom. The color scheme in the heatmap corresponds to the scalar density values at each pixel.

We compared our picked virus particles with a popular AI-based particle-picking method, Topaz [22], considering factors such as the total number of classes, number of picked particles, 2D resolution, and visual orientation. Our manually picked particles have a better 2D class resolution than Topaz. It is noteworthy that a higher particle count alone does not ensure higher resolution. Instead, selecting a substantial number of high-quality particles across a broad angular distribution is crucial for achieving both high 2D and 3D resolution. The 2D class comparisons for two databases, EMPIAR 10205 and EMPIAR 10193 (each containing 1000 micrographs), are shown in Table 3 and Figure 7. In both cases, Topaz picked many more particles than CryoVirusDB but it had a worse weighted average resolution of 2D classes. The weighted average resolution of 2D classes is calculated by multiplying each class’s resolution by its particle count, summing these products, and dividing by the total number of particles across all classes.

We also assessed the density projections derived from the intermediate output during the ab initio reconstruction phase, as depicted at the bottom of each block in Figure 7. The plot illustrates the integrated density values along the perpendicular direction to that plane. The heatmap’s color scheme represents scalar density values at each pixel, with the intensity of color indicating the magnitude of density. This indicates the high quality of the virus particles in CryoVirusDB.

#### 3.3.2. 3D Density Map Validation

We reconstructed 3D density maps from the particles in CryoVirusDB and from those picked by Topaz for two datasets, EMPIAR 10205 and EMPIAR 10193, each comprising 1000 micrographs. The ab initio density map reconstruction and homogenous refinement were carried out in CryoSPARC using the generated star files that included the selected particles. To ensure an unbiased evaluation, we repeated the ab initio 3D reconstruction experiment with three distinct random seeds for each method.

**Figure 8:**
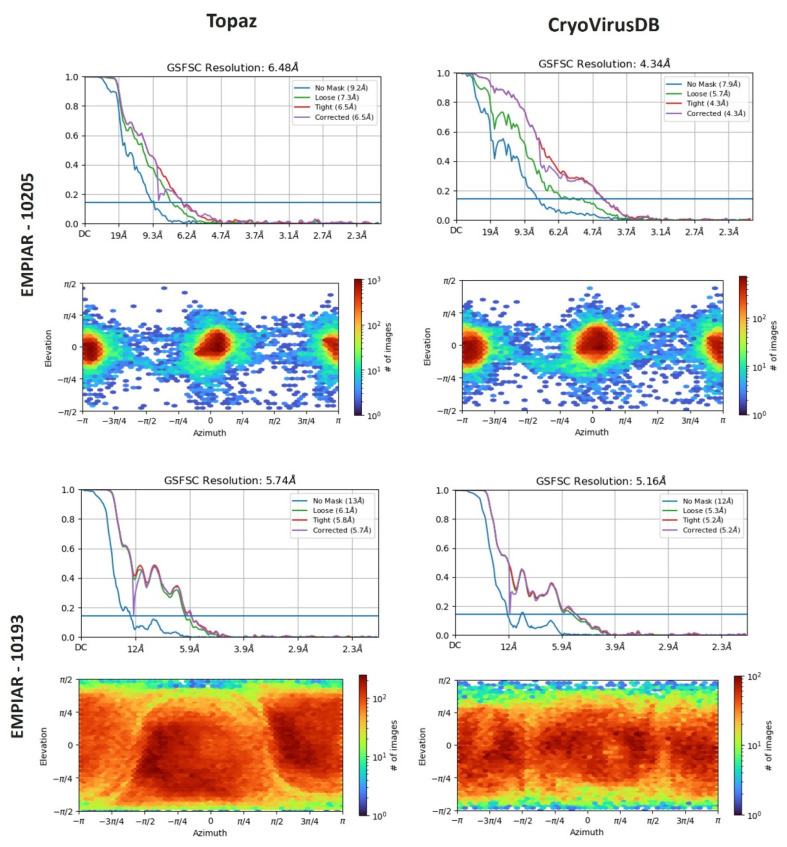
The comparison of reconstructed 3D density map resolution and direction distribution obtained by Topaz and CryoVirusDB on EMPIAR 10205 and EMPIAR 10193.

Figure 8 presents a comparison of the resolution and distribution direction of the reconstructed 3D density maps. The Fourier Shell Correlation (FSC) plots include a ‘loose mask’ curve that utilizes an automatically generated mask with a 15 Å falloff, and a ‘tight mask’ curve that employs an auto-generated mask with a falloff of 6 Å for all FSC plots.

In the case of EMPIAR 10205, Topaz picked around 75,000 more particles compared to our manual picking. Despite this, the density map reconstructed from particles selected by CryoVirusDB achieved a resolution of 4.34 Å, substantially better than Topaz’s resolution of 6.48 Å. This indicates the high quality of the picked particles in CryoVirusDB. For EMPIAR 10193, the resolution of CryoVirusDB is 5.16 Å, also better than 5.74 Å of Topaz.

The heightened intensity of the red color in the direction distribution shown in the lower section of each block in Figure 8 corresponds to an increased number of particles in the elevation vs. azimuth plots. CryoVirusDB demonstrated superior particle picking by capturing a substantial number of particles with a wide angular distribution, evident in the red coloration on the heatmap for both validation cases.

The detailed comparison of the 3D density map reconstruction of three trials for CryoVirusDB and Topaz is provided in Table 4. The density maps constructed from the labeled particles in CryoVirusDB consistently exhibit a higher quality than Topaz in terms of multiple resolution metrics, even though the number of particles in CryoVirusDB is much smaller than the number of particles picked by Topaz, indicating that Topaz may pick some false positives and/or miss some true positives representing different views.

### 3.4. Machine Learning Workflows with CryoVirusDB

#### 3.4.1. Denoising and Preprocessing Cryo-EM Micrographs

Micrographs in mrc format (micrographs directory) serve as input for AI models. To enhance signal-to-noise ratio, we recommend applying Gaussian filters for noise reduction. Standard normalization procedures should be applied to ensure consistent intensity distributions across micrographs using the formula [pixel = (pixel − μ)/σ], where pixel intensity is normalized relative to the mean (μ) and standard deviation (σ).

Subsequently, normalized micrographs should be converted to 8-bit grayscale, collapsing multi-channel intensity information into a single channel. This standardizes pixel values to a 0–255 range and reduces input size, minimizing computational load during model training. Various filtering and contrast enhancement techniques can be employed to further improve image quality. The preprocessing pipeline for denoising micrographs can be adapted from established resources [20,24,39].

#### 3.4.2. Generating Labels for Input Micrographs

The dataset includes expert-annotated particle coordinates within cryo-EM micrographs (ground_truth/particle_coordinates.csv). After preprocessing, corresponding labels must be generated for supervised learning using one of two widely adopted methods. First, coordinates can be stored in Common Objects in Context (COCO) format [40], a standard for object detection that structures labels and metadata in JSON format, enabling annotation of multiple particles within a single micrograph with category labels, bounding boxes, areas, micrograph references, and unique particle IDs (see Section 2.1.3 of CryoTransformer [20]). Alternatively, ground-truth segmentation masks can be generated from CSV coordinate data containing particle centers and diameters, serving as target labels during training for pixel-wise comparison between predicted and ground-truth masks for loss calculation, which is particularly effective for semantic segmentation tasks (refer to CryoSegNet [24] for detailed methodology).

#### 3.4.3. Machine Learning Model Development: Inputs and Expected Outputs

Supervised machine learning models—including convolutional neural networks (CNNs), transformers, artificial neural networks (ANNs), or segmentation models—can be designed to accurately predict bounding boxes of virus particles within cryo-EM micrographs while minimizing false positives. The input layer receives preprocessed micrographs, while the output layer predicts particle coordinates (X, Y).

These predicted coordinates are then used to generate .star files, which can be imported into widely used cryo-EM processing software packages such as CryoSPARC [35] or RELION [17] for further analysis and refinement.

## 4. Discussion and Conclusions

Here, we present a comprehensive dataset, CryoVirusDB, which offers researchers a rich dataset for training and testing AI and machine learning algorithms for virus particle identification from cryo-EM micrographs. With nearly 10,000 high-resolution cryo-EM micrographs and over 330,000 labeled virus particles spanning 9 datasets from 7 distinct non-enveloped icosahedral viruses, this database facilitates 3D reconstruction of virus structures from 2D particles, which is crucial for understanding viral structural mechanisms and developing targeted therapeutics.

CryoVirusDB uses curated subsets (around 1000 micrographs per EMPIAR ID) rather than complete datasets to balance computational feasibility with high-quality ground truth generation. While this reduces final map resolution compared to full-dataset reconstructions (4.3 Å vs. 2.7 Å for EMPIAR-10205 and 5.2 Å vs. 3.8 Å for EMPIAR-10193), our validation shows that manually curated particles consistently outperform automated picks (Topaz) at equivalent particle counts. This demonstrates that CryoVirusDB’s primary goal—providing superior training data for AI models—is achieved despite lower absolute resolution. Researchers can leverage models trained on our high-quality subsets to analyze complete datasets for maximum resolution.

While CryoVirusDB focuses on non-enveloped icosahedral viruses, the deep learning models trained on this dataset can potentially be extended to enveloped viruses through transfer learning approaches. Enveloped viruses such as influenza and SARS-CoV-2 present distinct challenges including pleomorphic morphology, ambiguous membrane boundaries, and flexible spike proteins. However, models pre-trained on CryoVirusDB capture fundamental cryo-EM image features—noise patterns, defocus variations, edge characteristics, and contamination signatures—that are independent of specific particle morphologies. These learned representations can be leveraged through fine-tuning on smaller annotated datasets of enveloped viruses. This transfer learning strategy would require substantially less manual annotation effort compared to training models from scratch, while enabling AI-based pickers to generalize across diverse viral architectures and advancing automated particle identification throughout structural virology.

## Figures and Tables

**Table 1:** The statistics of micrographs and particles of nine viruses in CryoVirusDB.

SN	EMPIAR ID	Virus Type	Number of Micrographs	Micrograph Size	Particle Diameter (px)	Defocus Range (μm)	Number of Virus Particles	Average Particles per Micrograph
1	10192 [26]	Feline calicivirus	1000	(4096, 4096)	470	−1.2 to −3.5	9660	9.66
2	11060 [27]	Nudaurelia capensis omega virus	1276	(4096, 4096)	516	−0.70 to −2.2	11,916	9.34
3	10203 [28]	Macrobrachium rosenbergii nodavirus	1000	(3838, 3710)	377	−1.0 to −2.5	16,601	16.60
4	10033 [29]	Human parechovirus 3	1000	(4096, 4096)	350	−0.42 to −2.34	55,732	55.73
5	10652 [30]	Coxsackievirus B4	1127	(3838, 3710)	374	−0.60 to −3.0	11,144	9.89
6	10341 [31]	Bovine enterovirus	1274	(4096, 4096)	376	−0.75 to −3.5	22,694	17.81
7	10193 [26]	Feline calicivirus	1000	(4096, 4096)	516	−1.2 to −3.5	96,126	96.13
8	10205 [32]	Cowpea mosaic virus	1000	(4096, 4096)	310	−0.50 to −3.5	81,037	81.04
9	10555 [33]	Nudaurelia capensis omega virus	1264	(4096, 4096)	564	−0.70 to −2.7	34,488	27.28
		Total	9941				339,398	34.14

**Table 2:** Metrics of EM data acquisition and grid preparation used in importing micrographs for virus particle picking.

SN	EMPIAR ID	Pixel Size (Å)	Electron Dose (e/Å^2^)	Detector
1	10192	1.065	63	FEI FALCON III (4k × 4k)
2	11060	1.065	46	FEI FALCON III (4k × 4k)
3	10203	1.06	36	GATAN K2 QUANTUM (4k × 4k)
4	10033	1.14	36	FEI FALCON II (4k × 4k)
5	10652	1.06	47	GATAN K2 SUMMIT (4k × 4k)
6	10341	1.065	49.5	FEI FALCON III (4k × 4k)
7	10193	1.065	63	FEI FALCON III (4k × 4k)
8	10205	1.065	67.5	FEI FALCON III (4k × 4k)
9	10555	1.0651	72	FEI FALCON III (4k × 4k)

Note: All datasets were acquired using an FEI TITAN KRIOS microscope (Thermo Fisher Scientific, Hillsboro, OR, USA) at 300 kV acceleration voltage with 2.7 mm spherical aberration. Micrographs are in mrc format.

**Table 3:** 2D classification result comparison for EMPIAR 10205 and EMPIAR 10193.

**EMPIAR 10205**		
	**2D Particle Class Statistics** **(Topaz)**	**2D Particle Class Statistics** **(CryoVirusDB)**
Number of Picked Particles	155,953	81,037
Weighted Average Resolution of 2D classes (*N* = 50)	9.41 Å	6.59 Å
Weighted Average Resolution of 2D classes (*N* = 10)	13.42 Å	10.96 Å
**EMPIAR 10193**		
	**2D Particle Class Statistics** **(Topaz)**	**2D Particle Class Statistics** **(CryoVirusDB)**
Number of Picked Particles	239,852	96,126
Weighted Average Resolution of 2D classes (*N* = 50)	18.52 Å	15.02 Å
Weighted Average Resolution of 2D classes (*N* = 10)	23.68 Å	21.72 Å

**Table 4:** 3D density map result comparison for EMPIAR 10205 and EMPIAR 10193. Bold fonts highlight the resolution of the best of the three trials for each method.

**EMPIAR 10205**						
	**3D Density Map Statistics** **(Topaz)**			**3D Density Map Statistics** **(CryoVirusDB)**		
Number of Picked Particles	155,953			81,037		
GSFSC Resolution (Å)	Trial 1	Trial 2	Trial 3	Trial 1	Trial 2	Trial 3
	6.97	6.59	**6.48**	**4.34**	4.40	4.47
No Mask Resolution (Å)	9.3	9.5	**9.2**	**7.9**	8.8	8.1
Loose Mask Resolution (Å)	7.7	7.2	**7.3**	**5.7**	7.1	6.3
Tight Mask Resolution (Å)	6.8	6.6	**6.5**	**4.3**	4.5	4.4
Corrected Mask Resolution (Å)	7	6.6	**6.5**	**4.3**	4.4	4.5
**EMPIAR 10193**						
	**3D Density Map Statistics** **(Topaz)**			**3D Density Map Statistics** **(CryoVirusDB)**		
Number of Picked Particles	239,852			96,126		
GSFSC Resolution (Å)	Trial 1	Trial 2	Trial 3	Trial 1	Trial 2	Trial 3
	5.86	**5.74**	5.82	**5.16**	5.22	5.18
No Mask Resolution (Å)	12	**13**	11	**12**	9.4	9.1
Loose Mask Resolution (Å)	5.9	**6.1**	5.8	**5.3**	5.8	5.5
Tight Mask Resolution (Å)	5.8	**5.8**	5.7	**5.2**	5.2	5.2
Corrected Mask Resolution (Å)	5.9	**5.7**	5.6	**5.2**	5.2	5.3

## Data Availability

The GitHub repository https://github.com/BioinfoMachineLearning/CryoVirusDB (accessed on 1 December 2025) provides the instructions on how to download and use the data. It also contains all of the scripts used in different stages of data curation. Particle coordinate annotations for CryoVirusDB are publicly available on Zenodo [25] at https://zenodo.org/records/10397742 (accessed on 1 December 2025).

## Supplementary Materials

The following supporting information can be downloaded at https://www.mdpi.com/article/10.3390/v18020224/s1. Table S1: Intermediate metadata associated with various steps of data curation.

## Abbreviations

The following abbreviations are used in this manuscript:

AI	Artificial Intelligence
ML	Machine Learning
CTF	Contrast Transfer Function
Cryo-EM	Cryogenic Electron Microscopy
CSV	Comma-Separated Values
EMDB	Electron Microscopy Data Bank
EMPIAR	Electron Microscopy Public Image Archive
FSC	Fourier Shell Correlation
GSFSC	Gold Standard Fourier Shell Correlation
NCC	Normalized Cross-Correlation
PDB	Protein Data Bank
PS	Power Spectrum
Å	Angstrom
kV	Kilovolt
e/Å^2^	Electron Dose per Square Angstrom
µm	Micrometer
